# Supplementary material for: HIVprotI: an integrated web based platform for prediction and design of HIV proteins inhibitors
Source: J Cheminform. 2018 Mar 9;10:12. doi: 10.1186/s13321-018-0266-y (PMC5845081; doi:10.1186/s13321-018-0266-y)
Supplement: Supplementary file 2 — Additional file 2. Source code of HIVProtI web server. [file 13321_2018_266_MOESM2_ESM.zip › HIVprotI_Source-code/hivprotI/weka-3-6-6/doc/weka/core/Debug.Clock.html]

Debug.Clock


---


|  |  |  |
| --- | --- | --- |
| **Overview** \| **Package** \| **Class** \| **Tree** \| **Deprecated** \| **Index** \| **Help** \| **Weka's home** | |  |
| **PREV CLASS**   **NEXT CLASS** | **FRAMES**    **NO FRAMES**     **All Classes** |  |
| SUMMARY: NESTED \| FIELD \| CONSTR \| METHOD | DETAIL: FIELD \| CONSTR \| METHOD |  |


---


## weka.core Class Debug.Clock

```
java.lang.Object
  weka.core.Debug.Clock
```

**All Implemented Interfaces:**: java.io.Serializable, RevisionHandler

**Enclosing class:**: Debug

---

``` public static class Debug.Clock extends java.lang.Object implements java.io.Serializable, RevisionHandler ```

A little helper class for clocking and outputting times. It measures the
CPU time if possible, otherwise it's just based on the system time. In
case one just wants to measure time (e.g., database queries don't take up
much CPU time, but still might take a long time to finish), then one can
disable the use of CPU time as well.

**Version:**
:   $Revision: 7519 $

**Author:**
:   FracPete (fracpete at waikato dot ac dot nz)

**See Also:**: `ThreadMXBean.isThreadCpuTimeEnabled()`, Serialized Form

---

| **Field Summary** | |
| --- | --- |
| `static int` | `FORMAT_HHMMSS`             the output format in hours:minutes:seconds, with fraction of msecs |
| `static int` | `FORMAT_MILLISECONDS`             the output format in milli-seconds |
| `static int` | `FORMAT_SECONDS`             the output format in seconds, with fraction of msecs |
| `int` | `m_OutputFormat`             the format of the output |
| `static Tag[]` | `TAGS_FORMAT`             the output formats |


**Constructor Summary**

`Debug.Clock()`
  
          automatically starts the clock with FORMAT\_SECONDS format and CPU
time if available

`Debug.Clock(boolean start)`
  
          starts the clock depending on `start` immediately with the
FORMAT\_SECONDS output format and CPU time if available

`Debug.Clock(boolean start,
int format)`
  
          starts the clock depending on `start` immediately, using
CPU time if available

`Debug.Clock(int format)`
  
          automatically starts the clock with the given output format and CPU
time if available


| **Method Summary** | |
| --- | --- |
| `int` | `getOutputFormat()`             returns the output format |
| `java.lang.String` | `getRevision()`             Returns the revision string. |
| `long` | `getStart()`             returns the start time |
| `long` | `getStop()`             returns the stop time or, if still running, the current time |
| `boolean` | `getUseCpuTime()`             returns whether the use of CPU is time is enabled/disabled (regardless whether the system supports it or not) |
| `boolean` | `isCpuTime()`             whether the measurement is based on the msecs returned from the System class or on the more accurate CPU time. |
| `boolean` | `isRunning()`             whether the time is still being clocked |
| `void` | `setOutputFormat(int value)`             sets the format of the output |
| `void` | `setUseCpuTime(boolean value)`             enables/disables the use of CPU time (if measurement of CPU time is available). |
| `void` | `start()`             saves the current system time (or CPU time) in msec as start time |
| `void` | `stop()`             saves the current system (or CPU time) in msec as stop time |
| `java.lang.String` | `toString()`             returns the elapsed time, getStop() - getStart(), as string |

**Methods inherited from class java.lang.Object**

`equals, getClass, hashCode, notify, notifyAll, wait, wait, wait`

**Field Detail**

### FORMAT\_MILLISECONDS

```
public static final int FORMAT_MILLISECONDS
```

:   the output format in milli-seconds

    **See Also:**: Constant Field Values

---


### FORMAT\_SECONDS

```
public static final int FORMAT_SECONDS
```

:   the output format in seconds, with fraction of msecs

    **See Also:**: Constant Field Values

---


### FORMAT\_HHMMSS

```
public static final int FORMAT_HHMMSS
```

:   the output format in hours:minutes:seconds, with fraction of msecs

    **See Also:**: Constant Field Values

---


### TAGS\_FORMAT

```
public static final Tag[] TAGS_FORMAT
```

:   the output formats

---


### m\_OutputFormat

```
public int m_OutputFormat
```

:   the format of the output


**Constructor Detail**

### Debug.Clock

```
public Debug.Clock()
```

:   automatically starts the clock with FORMAT\_SECONDS format and CPU
    time if available

    **See Also:**: `m_OutputFormat`

---


### Debug.Clock

```
public Debug.Clock(int format)
```

:   automatically starts the clock with the given output format and CPU
    time if available

    **Parameters:**: `format` - the output format **See Also:**: `m_OutputFormat`

---


### Debug.Clock

```
public Debug.Clock(boolean start)
```

:   starts the clock depending on `start` immediately with the
    FORMAT\_SECONDS output format and CPU time if available

    **Parameters:**: `start` - whether to start the clock immediately **See Also:**: `m_OutputFormat`

---


### Debug.Clock

```
public Debug.Clock(boolean start,
                   int format)
```

:   starts the clock depending on `start` immediately, using
    CPU time if available

    **Parameters:**: `start` - whether to start the clock immediately: `format` - the format **See Also:**: `m_OutputFormat`


**Method Detail**

### isCpuTime

```
public boolean isCpuTime()
```

:   whether the measurement is based on the msecs returned from the System
    class or on the more accurate CPU time. Also depends on whether the
    usage of the CPU time was disabled or enabled.

    :   **Returns:**: true if the more accurate CPU time of the thread is used and the use of CPU time hasn't been disabled **See Also:**: `System.currentTimeMillis()`, `ThreadMXBean.isThreadCpuTimeEnabled()`, `getUseCpuTime()`

---


### setUseCpuTime

```
public void setUseCpuTime(boolean value)
```

:   enables/disables the use of CPU time (if measurement of CPU time is
    available). The actual use of CPU time still depends on whether the
    system supports it. Resets the current timer, if running.

    :   **Parameters:**: `value` - if true the CPU time is used (if possible)

---


### getUseCpuTime

```
public boolean getUseCpuTime()
```

:   returns whether the use of CPU is time is enabled/disabled (regardless
    whether the system supports it or not)

    :   **Returns:**: true the CPU time is used (if possible)

---


### start

```
public void start()
```

:   saves the current system time (or CPU time) in msec as start time

    :   **See Also:**: `m_Start`

---


### stop

```
public void stop()
```

:   saves the current system (or CPU time) in msec as stop time

    :   **See Also:**: `m_Stop`

---


### getStart

```
public long getStart()
```

:   returns the start time

    :   **Returns:**: the start time

---


### getStop

```
public long getStop()
```

:   returns the stop time or, if still running, the current time

    :   **Returns:**: the stop time

---


### isRunning

```
public boolean isRunning()
```

:   whether the time is still being clocked

    :   **Returns:**: true if the time is still being clocked

---


### setOutputFormat

```
public void setOutputFormat(int value)
```

:   sets the format of the output

    :   **Parameters:**: `value` - the format of the output **See Also:**: `m_OutputFormat`

---


### getOutputFormat

```
public int getOutputFormat()
```

:   returns the output format

    :   **Returns:**: the output format **See Also:**: `m_OutputFormat`

---


### toString

```
public java.lang.String toString()
```

:   returns the elapsed time, getStop() - getStart(), as string

    :   **Overrides:**: `toString` in class `java.lang.Object`
    :   **Returns:**: the elapsed time as string **See Also:**: `getStart()`, `getStop()`

---


### getRevision

```
public java.lang.String getRevision()
```

:   Returns the revision string.

    :   **Specified by:**: `getRevision` in interface `RevisionHandler`
    :   **Returns:**: the revision


---


|  |  |  |
| --- | --- | --- |
| **Overview** \| **Package** \| **Class** \| **Tree** \| **Deprecated** \| **Index** \| **Help** \| **Weka's home** | |  |
| **PREV CLASS**   **NEXT CLASS** | **FRAMES**    **NO FRAMES**     **All Classes** |  |
| SUMMARY: NESTED \| FIELD \| CONSTR \| METHOD | DETAIL: FIELD \| CONSTR \| METHOD |  |


---
